# Non-utilization of health facility delivery and its correlates among childbearing women: a cross-sectional analysis of the 2018 Guinea demographic and health survey data

**DOI:** 10.1186/s12913-020-05893-0

**Published:** 2020-11-09

**Authors:** Bright Opoku Ahinkorah

**Affiliations:** School of Public Health, Faculty of Health, University of Technology Sydney, Sydney, New South Wales Australia

**Keywords:** Home delivery, Childbearing women, Guinea, Community health, Global Health

## Abstract

**Background:**

Many childbearing women in sub-Saharan African countries like Guinea still face challenges accessing and utilizing health facility delivery services and opt to deliver at home. This study examined the non-utilization of health facility delivery and its associated factors among childbearing women in Guinea.

**Methods:**

Data from the 2018 Guinea Demographic and Health Survey was used in this study. Data of 5406 childbearing women were analysed using STATA version 14.2 by employing a multilevel logistic regression approach. The results were presented using adjusted odds ratios (aOR) at 95% confidence interval (CI).

**Results:**

More than three-quarters (47.6%) of childbearing women in Guinea did not deliver at health facilities. Women who had no formal education (aOR = 1.52, 95% CI = 1.09–2.12), those whose partners had no formal education (aOR = 1.25, 95% CI =1.01–1.56), those whose pregnancies were unintended (aOR = 1.40, 95% CI =1.13–1.74) and those who were Muslims (aOR = 2.87, 95% CI =1.17–7.08) were more likely to deliver at home. Furthermore, women with parity four or more (aOR = 1.78, 95% CI =1.34–2.37), those who listened to radio less than once a week (aOR = 5.05, 95% CI =1.83–13.89), those who never watched television (aOR = 1.46, 95% CI =1.12–1.91), those with poorest wealth quintile (aOR = 4.29, 95% CI =2.79–6.60), women in female-headed households (aOR = 1.38, 95% CI =1.08–1.78) and rural dwellers (aOR = 3.86, 95% CI =2.66–5.60) were more likely to deliver at home.

**Conclusion:**

This study has identified low socio-economic status, inadequate exposure to media, having an unplanned pregnancy and religious disparities as key predictors of home delivery among childbearing women in Guinea. The findings call for the need to enhance advocacy and educational strategies like focus group discussions, peer teaching, mentor-mentee programmes at both national and community levels for women to encourage health facility delivery. There is also the need to improve maternal healthcare services utilization policies to promote access to health facility delivery by reducing costs and making health facilities available in communities.

## Background

Maternal mortality continues to be a key public health issue of great concern globally [1–3]. Globally, maternal mortality ratio as at 2019 stood at 211 per 100,000 live births [3]. Health facility deliveries have been found to be associated with reduction in maternal and newborn deaths [4, 5]. One of the key strategies recommended by the World Health Organization (WHO) in its attempt to reduce maternal and infant death is the accessibility of health facilities with skilled birth attendants who can deal with emergency obstetric cases [6].

In sub-Saharan Africa (SSA), the current maternal mortality ratio is 534 per 100,000 live births, which is higher than the average ratio globally (211 per 100,000 live births) [3]. Despite significant efforts to reduce maternal deaths through enhanced maternal healthcare services utilization globally [7–9], many childbearing women in SSA still face challenges accessing and utilizing maternal healthcare services, including delivery services and opt to deliver at home [10, 11].

Evidence suggests that home births pose high risks to the health of the mother and the child during the period after delivery [12, 13]. Some of these risks include desertion of colostrum provision and breastfeeding practices. Other risks include neglect of immunisations and nutrition supplementation for mother and child and lack of postnatal care check-up for the child and mother [14–16].

As part of efforts to enhance health facility delivery and reduce maternal and child mortality in Guinea, the Government introduced the Maternal Deaths Surveillance and Response (MDSR) which focused on carrying out routine exercises to identify, notify, quantify, and determine the causes of maternal deaths and how they can be avoided as well as providing education on the use of maternal healthcare services to mothers. This initiative was enhanced by setting up an online approach called “District.Team” in 2016, to facilitate horizontal learning between health district management teams (HDMTs) [17].

Despite all these efforts, Guinea continues to be one of the countries in SSA with high maternal mortality ratio as the country recorded 747 maternal deaths per 100,000 live births in 2010, then 699 per 100,000 live births in 2015 and 576 per 100,000 live births in 2017 [18]. The high maternal mortality ratio in Guinea has been attributed to the high home births due to several socio-cultural factors including having high trust and comfort in local birth attendants and community midwives, distance to health facilities, and inadequate financial resources to cater for transportation cost and other costs that are not catered for during delivery [19]. This to some extent has also contributed to the high under-five mortality recorded in the country. For instance, in 2018, child mortality rate for Guinea was 101.1 deaths per 1000 live births [20].

Apart from these socio-cultural factors, studies conducted in different sub-Saharan African countries such as Nigeria [21, 22], Guinea-Bissau [23], Zambia [24], Tanzania [25], Kenya [26] and Ghana [27] have found individual and contextual level factors such as maternal age, maternal level of education, marital status, place of residence, exposure to media, religion, parity, wealth status and women’s decision making capacity as predictors of home delivery.

Despite the high rate of home delivery with its associated maternal and child mortality in Guinea, there has not been any study that has examined the factors associated with the non-utilization of health facility delivery in the country. This study, therefore, examined the factors associated with the non-utilization of health facility delivery among childbearing women in Guinea using data from the 2018 Guinea Demographic and Health Survey. Findings from the study are important to Guinea as they can form the basis for improved health facility delivery services utilization, which can help reduce maternal and child mortality in the country.

## Methods

### Study area

The area for the study was Guinea. The Republic of Guinea is located in West Africa and is one of the world’s poorest countries, ranked 178th out of 187 countries on the 2011 Human Development Index [28]. Based on the 2019 population of 12.77 million people, Guinea’s population density is 134.5 people per square mile (51.9 people per square kilometer), which ranks 127th in the world [29]. According to the Integrated Core Survey for the Evaluation of Poverty (EIBEP 2002–2003), access to healthcare services in the country (under 30 min) was 38.9% and utilization of healthcare services was 18.6% [30].

### Study design and data source

A cross-sectional study design using data from the women’s file of the 2018 Guinea Demographic and Health Survey (GDHS) was considered in this study. GDHS is part of a number of surveys obtained from the MEASURE DHS Program and contains information on a number of population and health issues including place of delivery. In sampling respondents for the GDHS, a two-stage stratified sampling approach was employed. The first stage involved the selection of clusters usually called enumeration areas (EAs) and the second stage consisted of the selection of households for the survey. The average size of EAs was 186 household in urban areas and 140 households in rural areas, with an average of 153 households. Out of these households, 10,874 women were interviewed [31]. In this study, data of 5406 women aged 15–49 with at least one child ever born were analysed. Detailed information about the methods used in the survey can be found in the final report [31].

### Study variables

#### Outcome variable

Place of delivery was the outcome variable in this study. This was grouped into home and health facility delivery. Home delivery was described as any birth that took place in the women’s home or others’ home whereas health facility delivery referred to deliveries that took place in governmental health posts, health centers, hospitals, private clinics and maternity homes [31].

#### Explanatory variables

Fifteen explanatory variables, made up of 11 individual level factors (age, mother’s education level, partner’s educational level, pregnancy intention, religion, marital status, parity, employment status, frequency of reading newspaper, frequency of listening to radio, frequency of watching television) and four contextual factors (wealth quintile, place of residence, decision making in healthcare, and sex of household head) were considered for this study. Age was coded as 15–24, 25–34, and 35+. Mother and partner’s level of education were categorised into no education, primary and secondary/higher. Parity was coded as one birth, two births, three births and four or more births. Employment status was coded as working and not working. Not at all, less than once a week and at least once a week were used as codes for frequency of reading newspaper, listening to radio, and watching television. Wealth quintile was coded as poorest, poorer, middle, richer and richest. Place of residence was coded as rural and urban. Healthcare decision making was coded as alone and not alone and male and female were used as description for sex of household head. These variables were chosen due to their significant associations with non-utilization of health facility delivery in previous studies in SSA [12, 32–34].

### Statistical analyses

Analyses were carried out using a two-step analytical approach with the help of STATA version 14.2 for windows. First, the prevalence of non-utilization of health facility delivery and its distribution across the individual and contextual factors were presented. Statistical significance of the association between each of the factors and non-use of health facility delivery was measured using Pearson’s’ chi-square [χ^2^] at a *p* < 0.05 (see Table 1). This was followed by a two-level multilevel multivariable logistic regression analysis carried out to examine the individual and contextual factors associated with non-utilization of health facility delivery. Four models were fitted (Model 0, 1, 2 and 3) using the STATA command “melogit”. Models were compared using the log-likelihood ratio (LLR) and Akaike’s Information Criterion (AIC) tests and the model with the highest log-likelihood and the lowest AIC was regarded as the best fit model (see Table 2). Adjusted odds ratios (aOR) were presented for all the models apart from model 0 at 95% confidence intervals (CIs) (see Table 2). Multicollinearity test was done using the variance inflation factor (VIF) and no evidence of high collinearity was found. All frequency distributions were weighted. The manuscript was written by following the Strengthening Reporting of Observational studies in Epidemiology (STROBE) reporting guidelines [35].

**Table 1:** Distribution of non-utilization of health facility delivery by socio-demographic characteristics of childbearing women in Guinea (Weighted *N* = 5406)

Variables	Frequency (N)	Percentage (%)	Home delivery (47.6%)	***p***-value
**Age**				*p* = 0.001
15–24	1362	27.0	46.5	
25–34	2308	45.7	48.9	
35+	1376	27.3	53.6	
**Mother’s level of education**				*p* < 0.001
No education	3945	78.2	55.7	
Primary	531	10.5	39.9	
Secondary/Higher	570	11.3	15.8	
**Partner’s level of education**				*p* < 0.001
No education	3690	73.1	56.4	
Primary	357	7.1	42.8	
Secondary/Higher	999	19.8	25.7	
**Religion**				*p* < 0.001
Christianity	516	10.2	28.0	
Islam	4447	88.1	51.9	
Others	83	1.6	22.5	
**Pregnancy intention**				*p* < 0.001
Unintended	719	14.2	56.9	
Planned	4327	85.8	48.3	
**Marital status**				*p* < 0.001
Married	4944	98.0	49.9	
Cohabiting	102	2.0	29.8	
**Parity**				*p* < 0.001
One birth	785	15.6	37.9	
Two births	914	18.1	46.4	
Three births	952	18.9	47.8	
Four or more births	2395	47.5	55.1	
**Employment status**				*p = 0.082*
Not working	1252	24.8	47.5	
Working	3793	75.2	50.3	
**Frequency of reading newspaper**				*p* < 0.001
Not at all	4784	94.8	51.4	
Less than once a week	147	2.9	15.4	
At least once a week	116	2.3	7.1	
**Frequency of listening to radio**				*p* < 0.001
Not at all	2084	41.3	55.8	
Less than once a week	1402	27.8	42.8	
At least once a week	1560	30.9	47.2	
**Frequency of watching television**				*p* < 0.001
Not at all	3127	62.0	62.1	
Less than once a week	906	18.0	35.2	
At least once a week	1013	20.1	21.4	
**Wealth quintile**				*p* < 0.001
Poorest	1213	24.0	76.7	
Poorer	1139	22.6	62.0	
Middle	997	19.8	50.6	
Richer	956	19.0	26.0	
Richest	741	14.7	13.1	
**Sex of household head**				*p* < 0.001
Male	4525	88.7	50.7	
Female	521	10.3	39.6	
**Health care decision making**				*p = 0.016*
Alone	514	10.2	44.2	
Not alone	4532	89.8	50.1	
**Place of residence**				*p* < 0.001
Urban	1407	27.9	17.2	
Rural	3639	72.1	62.1	

Source: 2018 Guinea Demographic and Health Survey

**Table 2:** Predictors of non-utilization of health facility delivery among childbearing women in Guinea

Variables	Model 0	Model 1 aOR (CI)	Model 2 aOR (CI)	Model 3 aOR (CI)
**Age**				
15–24		Ref		Ref
25–34		0.80 (0.64–1.00)		0.89 (0.71–1.11)
35+		0.76^*^(0.58–0.99)		0.86 (0.65–1.12)
**Mother’s level of education**				
No education		1.88^***^(1.35–2.62)		1.52^*^(1.09–2.12)
Primary		1.39 (0.96–2.01)		1.28 (0.89–1.86)
Secondary/Higher		Ref		Ref
**Partner’s level of education**				
No education		1.49^***^(1.19–1.85)		1.25^*^(1.01–1.56)
Primary		1.16 (0.83–1.62)		1.05 (0.75–1.46)
Secondary/Higher		Ref		Ref
**Pregnancy intention**				
Unintended		1.36^**^(1.10–1.68)		1.40^**^(1.13–1.74)
Planned		Ref		Ref
**Religion**				
Christianity		0.72 (0.28–1.83)		0.96 (0.40–2.33)
Islam		1.36 (0.52–3.58)		2.87^*^(1.17–7.08)
Others		Ref		Ref
**Marital status**				
Married		1.08 (0.58–2.01)		1.09 (0.59–2.02)
Cohabiting		Ref		Ref
**Parity**				
One birth		Ref		Ref
Two births		1.67^***^(1.28–2.18)		1.59^***^(1.22–2.07)
Three births		1.60^***^(1.21–2.13)		1.51^**^(1.14–2.01)
Four or more births		2.00^***^(1.50–2.65)		1.78^***^(1.34–2.37)
**Frequency of reading newspaper**				
Not at all		5.26^***^(2.15–12.88)		4.52^***^(1.87–10.90)
Less than once a week		5.53^***^(1.98–15.45)		5.05^**^(1.83–13.89)
At least once a week		Ref		Ref
**Frequency of listening to radio**				
Not at all		0.97 (0.79–1.19)		0.99 (0.81–1.21)
Less than once a week		Ref		Ref
At least once a week		1.10 (0.90–1.35)		1.06 (0.87–1.31)
**Frequency of watching television**				
Not at all		2.42^***^(1.87–3.14)		1.46^**^(1.12–1.91)
Less than once a week		1.48^***^(1.13–3.14)		1.12 (0.85–1.49)
At least once a week		Ref		Ref
**Wealth quintile**				
Poorest			5.79^***^(3.84–8.72)	4.29^***^(2.79–6.60)
Poorer			3.72^***^(2.50–5.54)	2.82^***^(1.86–4.28)
Middle			2.54^***^(1.73–3.73)	1.93^**^(1.30–2.89)
Richer			1.58^***^(1.14–2.18)	1.28 (0.92–1.78)
Richest			Ref	Ref
**Sex of household head**				
Male			1.47^***^(1.15–1.88)	1.38^*^(1.08–1.78)
Female			Ref	Ref
**Health care decision making**				
Alone			Ref	Ref
Not alone			1.10 (0.85–1.41)	1.20 (0.93–1.55)
**Place of residence**				
Urban			Ref	Ref
Rural			4.92^***^(3.34–7.25)	3.86^***^(2.66–5.60)
**Random effects results**				
PSU Variance (95% CI)	2.0 (0.8–5.0)	2.2 (1.0–5.3)	2.1 (0.9–0.51)	2.1 (0.9–5.3)
ICC	0.51	0.39	0.30	0.25
LR Test	χ^2^ = 1394.0, *p* < 0.001	χ^2^ = 608.8, *p* < 0.001	χ^2^ = 477.68, *p* < 0.001	χ^2^ = 299.3, *p* < 0.001
Wald χ^2^	Reference	182.3^***^	347.1^***^	476.7^***^
Model fitness				
Log-likelihood	− 2800.5	− 2699.6	− 2651.4	−2588.1
AIC	5604.9	5441.2	5320.8	5232.3
Sample size	5406	5406	5406	5406

Source: 2018 Guinea Demographic and Health Survey

Model 0 is the null model, a baseline model without any determinant variable

Model 1 is adjusted for individual-level variables

Model 2 is adjusted for contextual-level variables

Model 3 is the final model adjusted for individual and contextual-level variables

*aOR* Adjusted odds ratios

*CI* Confidence interval

*Ref* Reference category

*PSU* Primary Sampling Unit

*ICC* Intra-Class Correlation

*LR Test* Likelihood ratio Test

*AIC* Akaike’s Information Criterion

^*^p < 0.05, ^**^p < 0.01, ^***^p < 0.001

### Ethical approval

This study made use of secondary data from DHS. Hence, no additional ethical approval was required as the data is freely available to the general public. However, the author sought permission from MEASURE DHS to use the data for this study. Details of the ethical standards are available on http://goo.gl/ny8T6X.

## Results

### Distribution of non-utilization of health facility delivery by socio-demographic characteristics of childbearing women in Guinea

Table 1 presents the results on the distribution of non-utilization of health facility delivery by socio-demographic characteristics of childbearing women in Guinea. Overall, 47.6% of childbearing women in Guinea delivered at home. It was found that 53.6% of the childbearing aged 35 years and above delivered at home, 55.7% of those who had no formal education and 56.4% of those whose partners had no formal education delivered at home. Home delivery was found to be high among Muslim women (51.9%), those who had unintended pregnancies (56.9%), married women (49.9%), women with four or more births (55.1%) and working women (50.3%). The majority of women who never read newspaper (55.4%), listened to radio (55.8%), watched television (62.1%), those with poorest wealth quintile (76.7%), those in male-headed households (50.7%), those who did not make healthcare decisions alone (50.1%) and those who lived in rural areas (62.1%) had high prevalence of home delivery. The chi-square analysis results also showed that all the independent variables, apart from employment status, showed statistically significant associations with home delivery (*p* < 0.05).

### Predictors of non-utilization of health facility delivery among childbearing women in Guinea

Table 2 shows the fixed and random effects results on the predictors of non-utilization of health facility delivery among childbearing women in Guinea. In terms of the fixed effects results, it was found that women who had no formal education (aOR = 1.52, 95% CI =1.09–2.12), those whose partners had no formal education (aOR = 1.25, 95% CI =1.01–1.56), those whose pregnancies were unintended (aOR = 1.40, 95% CI =1.13–1.74) and those who were Muslims (aOR = 2.87, 95% CI =1.17–7.08) had higher odds of delivery at home, compared to those who had secondary/higher education, those whose partners had secondary/higher education, those with planned pregnancies and those who belonged to other religions respectively. The results further showed that women with four or more births (aOR = 1.78, 95% CI =1.34–2.37), those who listened to radio less than once a week (aOR = 5.05, 95% CI =1.83–13.89), those who never watched television (aOR = 1.46, 95% CI =1.12–1.91), those with poorest wealth quintile (aOR = 4.29, 95% CI =2.79–6.60), women in male-headed households (aOR = 1.38, 95% CI =1.08–1.78) and those who lived in rural areas (aOR = 3.86, 95% CI =2.66–5.60) had higher odds of home delivery compared to those with one birth, those who read newspaper at least once a week, those who watched television at least once a week, those with richest wealth quintile, those in female-headed households and urban dwellers, respectively.

With the random effects results, the complete model (Model 3), which included all the individual and contextual level factors in the model and had an AIC of 5232.3 and a log-likelihood ratio of − 2588.1, was considered as the best fit model for predicting the occurrence of home delivery among childbearing women.

## Discussion

This study sought to assess the predictors of non-utilization of health facility delivery among childbearing women in Guinea. The prevalence of home delivery among childbearing women in Guinea is relatively high, as compared to what has been found previously in other countries in SSA like Ethiopia-44% [36] and Tanzania-21% [37]. Other studies, however, have identified high prevalence of home delivery, compared to this study. For instance, a study in Guinea found a prevalence of 61.8% home deliveries among women [38]. Other studies in Ghana [34], Nigeria [39] and Ethiopia [32], identified a prevalence of 59, 62 and 67.2% of home deliveries among women respectively. The disparities in prevalence of home delivery in this study, compared to other previous studies could be due to differences in study population and samples. The relatively high prevalence of home delivery among childbearing women in Guinea could be related to the disparities in access to maternal healthcare services, including health facility delivery in Guinea, which became very predominant during the Ebola outbreak and from which the country is still recovering [40–42]. The relatively high utilization of home delivery has implication for maternal healthcare utilization and maternal and child healthcare in Guinea. This is because, as many more pregnant women deliver at home, the use of health facility delivery which has been found to improve the health status of women and children will be low and this can lead to an increase in child and maternal mortality in the country.

Low socio-economic status, manifested in lack of formal education for both woman and partner, poorest wealth quintile and rural dwelling was linked with a higher likelihood of home delivery. Previous studies have also found factors such as lower wealth quintile [23, 34, 43, 44], lack of education [45–48] and rural dwelling [44, 49, 50] as predictors of home delivery. In most of these studies, the possible reasons provided for the increased likelihood of home delivery among these cohorts of pregnant women is financial and geographical barriers to accessing health facility deliveries. Other studies have also asserted that women who have no formal education, those whose partners have no formal education, those who are poor and live in rural areas may not have adequate knowledge about the risks associated with home delivery and hence may see no need to go and deliver at the health facility in the midst of their poor socio-economic status [12, 51, 52]. Findings on the association between socio-economic status and home delivery implies that enhancing health facility delivery will depend on improving the socio-economic status of pregnant women. This can be done through the collaborative efforts of government and non-governmental organisations in Guinea by providing women with skilled training and educational opportunities that will give them the economic empowerment to access health facility delivery.

In this study, women who were less exposed to media (newspaper and television) were more likely to deliver at home. Other previous studies have also identified a link between media exposure and choice of place of delivery, with women who are not exposed to media more likely to deliver at home compared to those who have media exposure [12, 53–55]. The possible reason for the increase in home delivery among women who are not exposed to media is that exposure to mass media offers high awareness and knowledge about pregnancy and birth-related complications [55]. It also changes a woman’s attitude, social norms and behavior that may lead to high access of health facility delivery [55]. The role of media in a pregnant woman’s choice of place of delivery calls for enhancement in access to media. Community-based information systems in Guinea can be used as platforms to communicate health messages to women while at the same time ensuring that women obtain the needed information they need from newspaper and television.

Muslim women were more likely to deliver at home in this study, compared to women who belonged to other religions. Similar to the findings of this study, a study in Nepal, identified that Muslim women were more likely to deliver at home compared to women of other religions [33]. A study in Ghana also identified low utilization of maternal healthcare skilled service among Muslim women [56]. The author explained that most Muslim women deliver at home because of the religious obligation to ensure a sanctified body through modest dressing and the avoidance of unlawful bodily exposure to people including male caregivers [56]. Other reasons were barriers to maternal healthcare such as lack of privacy, inadequate knowledge of healthcare workers about Muslim women’s religious and cultural practices and absence of cultural or religious-specific maternal healthcare services [56]. The high odds of home delivery among Muslim women could also be attributed to factors such as living in male-headed households and having four or more births, which were found to increase home deliveries and are sometimes considered as characteristics of Muslim women [57–59]. The findings on the association between religion and home delivery calls for the need for maternal healthcare services utilization strategies to pay more attention to Muslim women by exploring their religious norms and practices and understanding how they play a role in their choice of place of delivery.

Although unintended pregnancy has been considered to be associated with pregnancy-related complications such as poor weight gain, pregnancy-induced hypertension and anemia [60, 61], which warrants the need for health facility delivery, women with unintended pregnancies in this study had higher odds of delivering at home compared to those whose pregnancies were planned. This corroborates the findings of previous studies [62, 63]. The plausible reason for this finding can be understood in the context of age and unintended pregnancy, where studies have found high prevalence of unintended pregnancies among younger women [64–66]. Within this context, the high rate of home delivery attributed to unintended pregnancies could be explained in line with existing socio-cultural barriers and stigma which can hinder access to maternal healthcare services including health facility delivery [67–69]. The findings show the importance of encouraging pregnant women with unintended pregnancies to access health facility delivery by making them understand the implications of home delivery related to unintended pregnancies.

One key limitation of this study emanates from the design employed-cross sectional-which does not give room for causal inference to be made about its emergent results. Another limitation of the study is the difference in periods of data collection for the outcome variable “place of delivery” and the explanatory variables. Whiles data on the outcome variable asked questions on a phenomenon that occurred in the past, data on explanatory variables related to what was happening at the time of data collection, which may affect the findings and interpretations. In spite of these, the study presents current evidence on drivers of home delivery among childbearing women in Guinea using data from a nationally-representative survey.

## Conclusion

This study has identified factors such as low socio-economic status, low exposure to media, having an unplanned pregnancy and religious disparities as key predictors of non-utilization of health facility delivery among childbearing women in Guinea. The importance of enhancing health facility delivery and reducing if not eliminating home delivery among childbearing women cannot go unnoticed if Guinea can contribute in achieving target 1 of the Sustainable Development Goal 3 that aims to reduce global maternal mortality ratio to less than 70 per 100,000 live births by 2030. The findings calls for the need to enhance advocacy and educational strategies like focus group discussions, peer teaching, mentor-mentee programmes at both national and community levels for women to encourage health facility delivery. There is also the need to improve maternal healthcare services utilization policies to promote access to health facility delivery by reducing costs and making health facilities available in communities. It is important for future studies to employ qualitative design to provide a deeper understanding of some of the findings in the current study.

## Data Availability

Data for this study is available at: https://dhsprogram.com/data/dataset/Guinea_Standard-DHS_2018.cfm?flag=0.

## Abbreviations

AIC: Akaike’s Information Criterion
aOR: Adjusted odds ratios
CI: Confidence interval
GDHS: Guinea Demographic and Health Survey
ICC: Intra-Class Correlation
LR Test: Likelihood ratio Test
PSU: Primary Sampling Unit
SSA: Sub-Saharan Africa
